# First Intraoperative Trochlea Reconstruction

**DOI:** 10.1097/IOP.0000000000002498

**Published:** 2023-08-24

**Authors:** Stijn W. van der Meeren, Saskia P. A. van Hulst-Ginjaar, Nicoline E. Schalij-Delfos

**Affiliations:** * Department of Ophthalmology, Leiden University Medical Center, Leiden, The Netherlands; † Orbital Center, Department of Ophthalmology, Amsterdam University Medical Centers, Amsterdam, The Netherlands

## Abstract

Traumatic detachment of the superior oblique muscle from the trochlea is very rare. The authors present a case of cyclovertical diplopia in downgaze due to traumatic trochlear damage where they performed surgery more than 40 years later. For the first time ever, they describe the reconstruction of the trochlea using a silicone tube, thereby regaining superior oblique muscle function.

## Abstract 2

In a case of traumatic avulsion of the trochlea, intraoperative reconstruction of the trochlea using a silicone tube resolved the patient’s long-standing diplopia.

The trochlea is a ligamentous structure that ensures correct functioning of the superior oblique muscle. Damage to the trochlea may involve the muscle itself and/or can compromise the path of the muscle through the orbit. Reconstruction of the trochlea itself and obtaining gain of superior oblique muscle function has never been described. We present a case where we successfully created a trochlea from a silicone tube, illustrating the benefit of a surgeon’s intraoperative creativity. This study adhered to the tenets of the Declaration of Helsinki and the authors declare no conflicts of interest.

## CASE

As a third opinion, a 60-year-old male patient was referred for long-standing diplopia. His complaints started more than 40 years ago after a traumatic incident when a fishing hook damaged the superomedial part of his left orbit. Detailed old records could not be recovered but he had strabismus surgery 4 years after trauma. Ten years after the trauma, a 2 mm recession of the right inferior rectus was performed, followed by a recession of the left inferior oblique 1 year later.

**FIG. 1.**
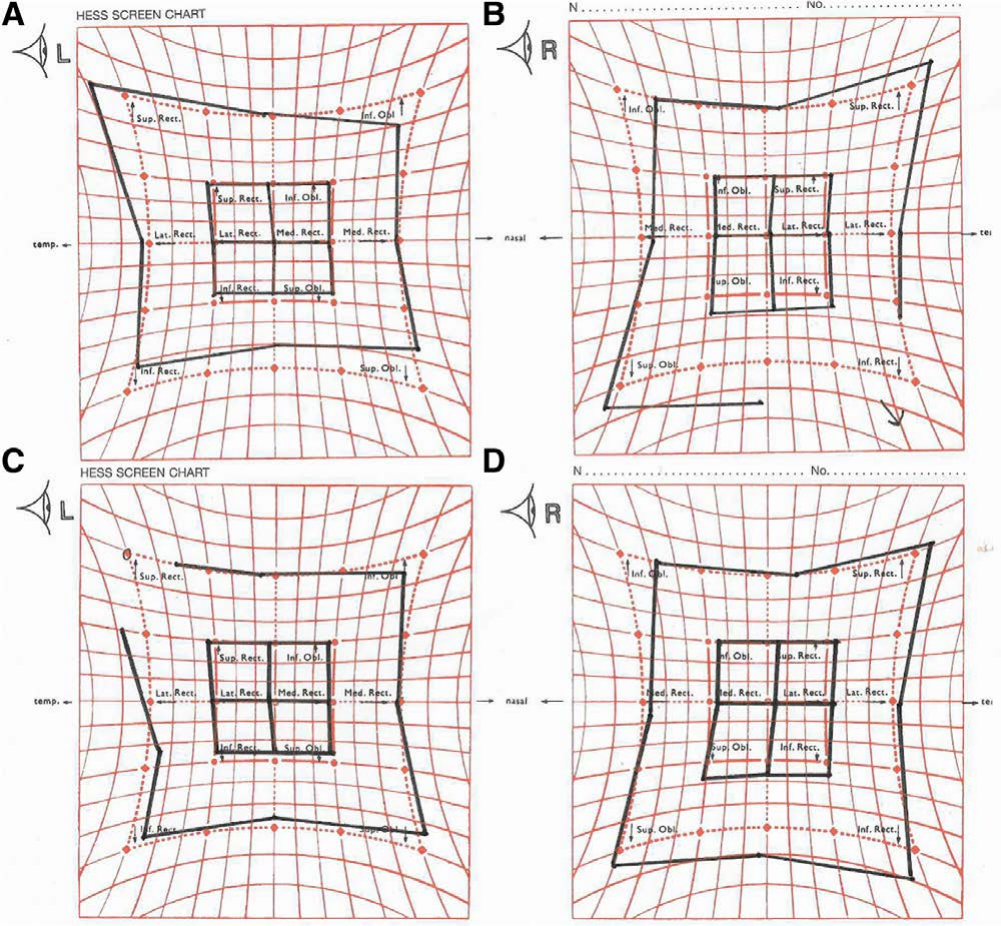
Hess screen charts preoperative (**A** and **B**) and postoperative (**C** and **D**).

Fourty years after the trauma, he was referred to our clinic for reading problems due to diplopia in downgaze. He had good visual acuity (1.5/1.5 Snellen) and normal findings on slit-lamp examination and fundoscopy. Orthoptic evaluation showed a compensating head tilt to the right with severe chin depression and intact stereoscopic vision. In primary position, without torticollis, he had 1° exo-, 1° left-hyperphoria, and 3° excyclophoria of the left eye. In downgaze, without torticollis, there was 5° hypertropia (left over right) and 7° excyclotropia of the left eye. The left over right increased in right and downgaze and the Parks-Bielchowsky 3 step test showed an increase of the left hypertropia on left head tilt. A 2− to 3− underaction of the left superior oblique muscle and 4+ overaction of the right inferior rectus muscle was found (Fig. 1). With the working diagnosis of a traumatic paresis of the left fourth cranial nerve, the patient was scheduled for a right inferior rectus muscle loop-recession and a tuck of the left superior oblique muscle. We expected this procedure to result in less torticollis and less diplopia in downgaze.

**FIG. 2.**
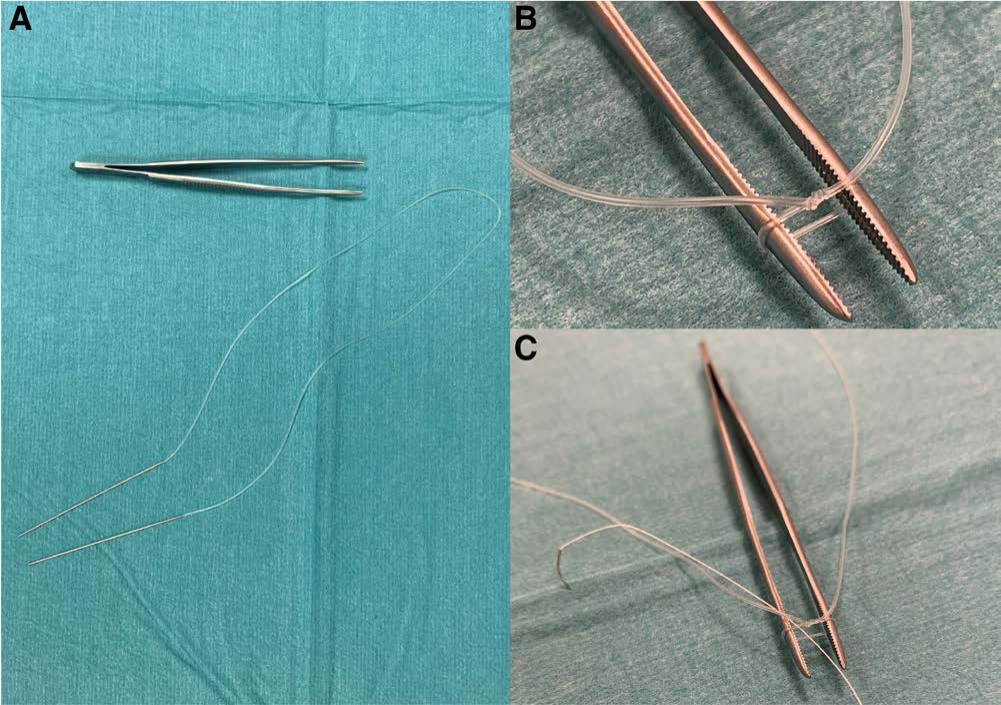
Used materials: simple forceps and a silicone tube, regularly used in lacrimal surgery, FCI BIKA 0.94 mm diameter (**A**); tube tied around the end of forceps with a square knot to create a small 3–4 mm silicone loop (**B**); a double-armed braided polyester 4-0 suture (Mersilene) was inserted through the loop (**C**).

**FIG. 3.**
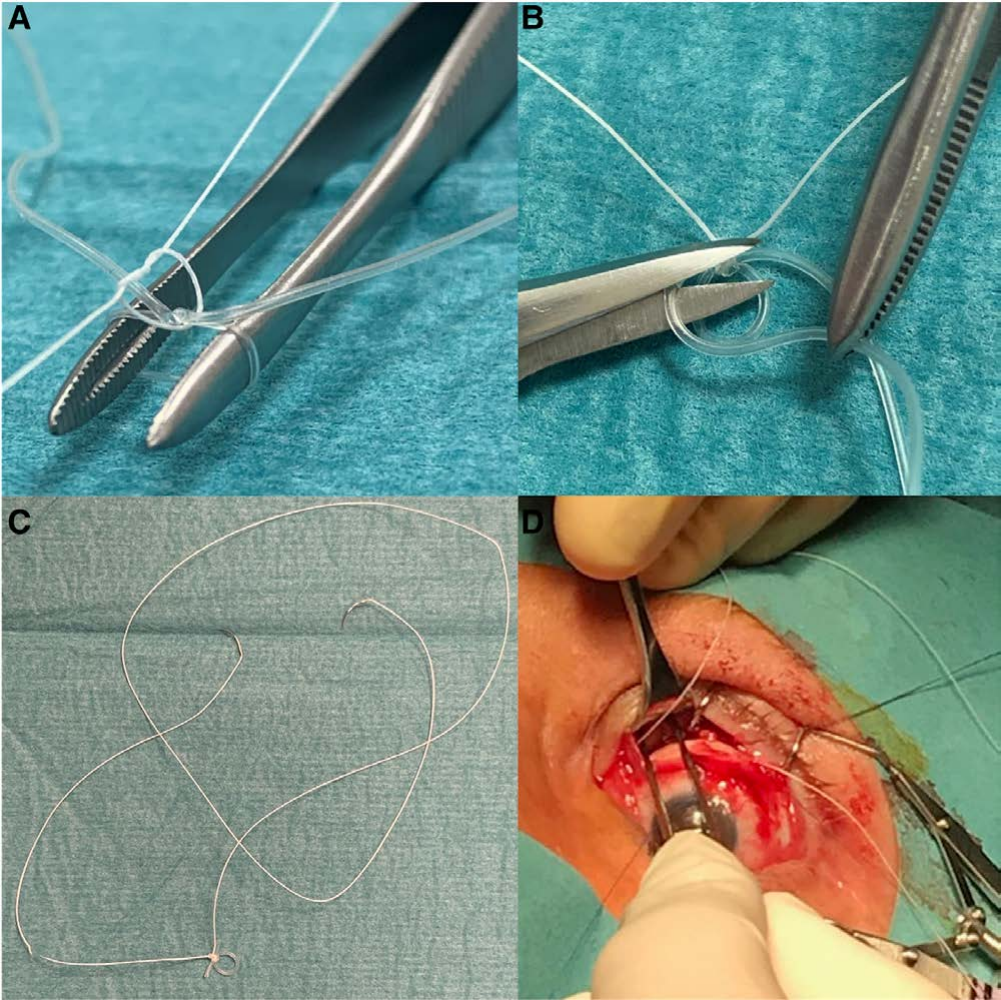
The polyester suture was tied around the silicon knot to secure the loop (**A**); silicone ends of the tube were cut (**B**); both needles of the suture were used (**C**); the silicone loop was attached a few millimeters posterior from the position of the original trochlea (**D**).

During the surgery, the superior oblique muscle was easily identified and was found not to follow its trajectory along the medial wall of the orbit but instead was running alongside the superior rectus muscle. Because the muscle was completely intact, we decided to try to reposition it to run along the medial part of the orbital roof by creating an artificial trochlea. A silicone tube, regularly used in lacrimal surgery (FCI BIKA, silicon, 0.94 mm diameter, Fig. 2A), was tied around the end of a forceps with a square knot to create a small silicone loop (Fig. 2B). A double-armed braided F2 polyester 4-0 suture (Mersilene) was tied around the knot (Fig. 2C) to secure the 3–4 mm silicone loop (Fig. 3A) and the silicone ends were cut (Fig. 3B). The contralateral (right) orbit was palpated to determine the position of the trochlea. When palpating the medial part of the anterior orbital roof of the affected (left) orbit, a very small bump indicated the position of the original trochlea. After gentle dissection, the silicone loop (Fig. 3C) was attached a few millimeters posteriorly from it using both ends of the double-armed suture (Fig. 3D). The superior F3 oblique muscle was then disinserted from its attachment to the globe, pulled through the silicone loop, and then re-attached to its original insertion with a 10 mm elongating Vicryl 6-0 loop suture.

One week after the surgery, the patient reported a tremendous improvement. After 4 months, he was able to read without diplopia and on examination there was only a slight residual head tilt toward the right shoulder, no changes in primary gaze and upper field, a minimal left-hyperphoria in downgaze, 1− under action of the left superior oblique and 1+ overaction of the right inferior rectus muscle (Fig. 1). After 4 years of follow-up, the patient still experiences no diplopia in his daily activities.

## DISCUSSION

This case is unique as it is the first description of a trochlea reconstruction using a silicon tube. Case reports on isolated damage of the superior oblique muscle are rare and reports on surgical treatment even more. Although eye surgeons will seldom encounter a partial or total detachment of the trochlea from the orbital wall, they should be aware that trochlea repair is possible. Of equal importance, this case stresses that creativity is an important quality in a surgeon.

In this case, we initially presumed the eye movement disorder to be the result of fourth cranial nerve palsy. Although this is not uncommonly caused by trauma, usually it is only seen in severe (closed) head trauma.^1,2^ The most common cause of trochlear damage is trochlea detachment following orbital roof fracture. Also, when repair of the orbital roof is necessary, the trochlea needs to be detached in order to allow for orbital roof repair. When the trochlea is still attached to the periosteum, reapproximation will suffice.^3^ The incidence of trochlear damage by surgical or accidental trauma is unknown but it has been reported to cause acquired Brown syndrome, whereas entrapment of the superior oblique muscle is rare.^4,5^

Some authors have described rupture of the superior oblique muscle, and superior oblique tendon damage can even result from eyelid surgery according to Kushner et al, who described 7 cases.^6,7^ In case of muscle or tendon rupture, reattachment of the muscle tendon or reapproximation of the tendon can have favorable results.^7,8^ If restoration of the superior oblique muscle is not possible, an ipsilateral inferior oblique recession can be combined with contralateral inferior rectus recession.^9^ In 1971, Dow described a case where muscle fibers were exposed through the conjunctiva and surgical repair of the tendon was performed by anchoring the tendon sheet to the medial orbital wall at the trochlea.^10^ Another case of exposed muscle fibers was described by Harish et al. In this case, no surgery was required, suggesting that the superior oblique muscle supposedly only needs a few fibers being properly attached and running correctly to eventually restore its functionality.^11^

We were unable to find any literature on an intact superior oblique muscle not running through the trochlea. Also, no literature reports are available that describe the reconstruction of a trochlea or the construction of a trochlea-like structure. In case of shortening of the muscle, a loop-recession from the original muscle insertion can be used for elongation to prevent a postoperative Brown syndrome. We are the first to show that in case of trochlear damage, or in case of rupture of the superior oblique muscle/tendon where the surgeon is unable to redirect the course of the muscle through the original trochlea, a simple silicone tube can be used to regain superior oblique muscle function.
